# Association between *MLH1* -93G>A Polymorphism and Risk of Colorectal Cancer

**DOI:** 10.1371/journal.pone.0050449

**Published:** 2012-11-30

**Authors:** Ting Wang, Yang Liu, Li Sima, Liang Shi, Zhaoming Wang, Chunhui Ni, Zhengdong Zhang, Meilin Wang

**Affiliations:** 1 Department of Environmental Genomics, Jiangsu Key Lab of Cancer Biomarkers, Prevention and Treatment, Cancer Center, Nanjing Medical University, Nanjing, P. R. China; 2 Department of Genetic Toxicology, the Key Laboratory of Modern Toxicology of Ministry of Education, School of Public Health, Nanjing Medical University, Nanjing, P. R. China; 3 The First Clinical Medical College, Nanjing Medical University, Nanjing, P. R. China; Ohio State University Medical Center, United States of America

## Abstract

**Background:**

The -93G>A (rs1800734) polymorphism located in the promoter of mismatch repair gene, *MLH1*, has been identified as a low-penetrance variant for cancer risk. Many published studies have evaluated the association between the *MLH1* -93G>A polymorphism and colorectal cancer (CRC) risk. However, the results remain conflicting rather than conclusive.

**Objective:**

The aim of this study was to assess the association between the *MLH1* -93G>A polymorphism and the risk of CRC.

**Methods:**

To derive a more precise estimation of the association, a meta-analysis of six studies (17,791 cases and 13,782 controls) was performed. Odds ratios (ORs) and 95% confidence intervals (CIs) were used to evaluate the strength of the association. Four of these published studies were performed on subjects of known microsatellite instability (MSI) status. An additional analysis including 742 cases and 10,895 controls was used to assess the association between the *MLH1* -93G>A polymorphism and the risk of MSI-CRC.

**Results:**

The overall results indicated that the variant genotypes were associated with a significantly increased risk of CRC (AG versus GG: OR = 1.06, 95% CI = 1.01–1.11; AA/AG versus GG: OR = 1.06, 95% CI = 1.01–1.11). This increased risk was also found during stratified analysis of MSI status (AA versus GG: OR = 2.52, 95% CI = 1.94–3.28; AG versus GG: OR = 1.29, 95% CI = 1.10–1.52; AA/AG versus GG: OR = 1.45, 95% CI = 1.24–1.68; AA versus AG/GG: OR = 2.29, 95% CI = 1.78–2.96). Egger’s test did not show any evidence of publication bias.

**Conclusion:**

Our results suggest that the *MLH1* -93G>A polymorphism may contribute to individual susceptibility to CRC and act as a risk factor for MSI-CRC.

## Introduction

Colorectal cancer (CRC) is the third most common cancer worldwide. There were over 1.2 million new cases and an estimated 608,700 deaths in 2008 alone [1]. Accumulating evidence suggests that CRC is caused by a set complex of interactions between environmental and genetic factors [2]. Deficiency in DNA mismatch repair (MMR) plays several important roles in the etiology of CRC. The MMR genes encode a family of highly conserved proteins, including MLH1, MSH2, MSH6, and PMS2 [3], [4]. MMR systems promote genetic stability by repairing DNA replication errors, inhibiting recombination between non-identical DNA sequences, and participating in responses to DNA damage [5]. DNA replication errors and mispairings cause microsatellite instability (MSI), a phenomenon frequently observed in sporadic CRC [6]. Rare constitutional mutations and methylation of *MLH1* and other MMR genes are the primary causes of the autosomal dominant disorder hereditary non-polyposis colorectal cancer (HNPCC) [7], [8]. MMR genes also contain common single nucleotide polymorphisms (SNPs) which can predispose individuals to sporadic CRC with low to moderate penetrance [9].

**Figure 1:**
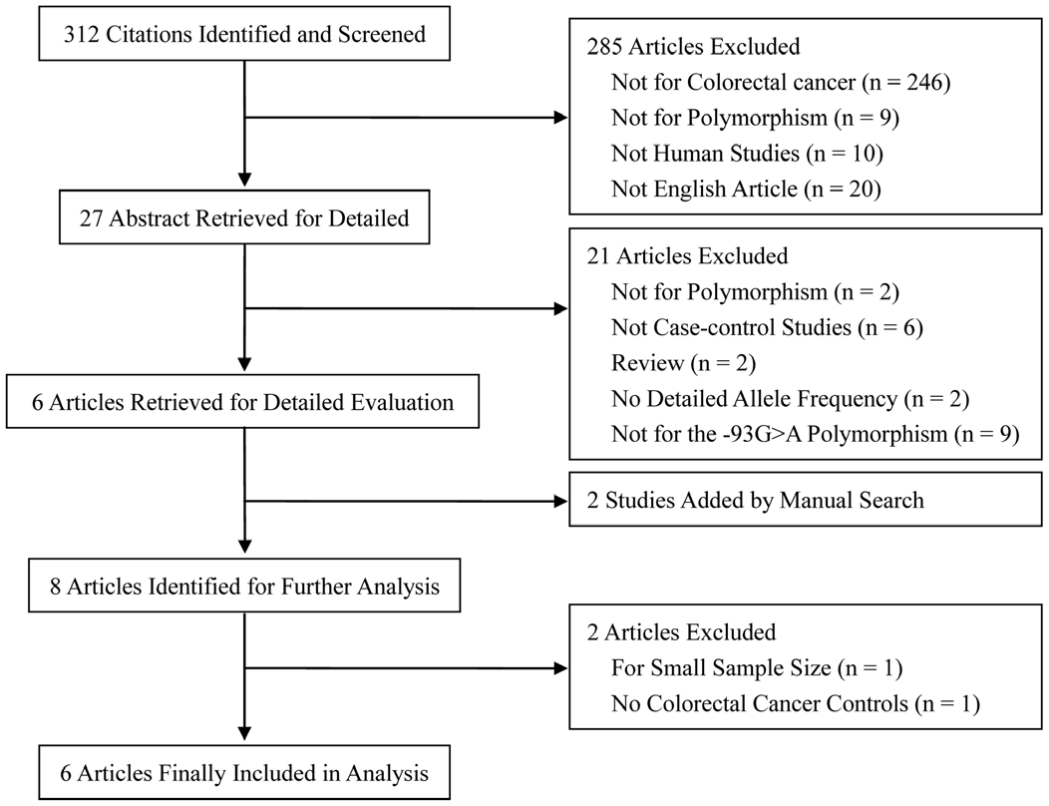
Articles identified with criteria for inclusion and exclusion.

The -93G>A (rs1800734) polymorphism is located in the promoter region of *MLH1*, which is responsible for maximal transcriptional activity of this gene [10], [11]. Although the association between the *MLH1* -93G>A polymorphism and CRC risk has been demonstrated in several studies, results remain inconsistent. This may be partially due to the relatively small sample size evaluated in each study. To estimate the overall risk of the *MLH1* -93G>A polymorphism associated with CRC risk and to quantify potential inter-study heterogeneity, we conducted a meta-analysis on six published case-control studies with a total of 17,791 CRC cases and 13,782 controls.

**Table 1:** Characteristics of studies included in the CRC meta-analysis.

First author	Year	Country	Ethnicity	Source of controls	Sample size (case/control)	Genotyping method	Matching criteria	CRC MSI status (case/control)	HWE (controls)
Allan	2008	UK	European	Population	1518/589	PCR-RFLP	Age, sex	44/589	0.750
Koessler	2008	UK	European	Population	2288/2276	TaqMan	Age, residence area	–	0.914
Tulupova	2008	Czech	European	Hospital	609/611	TaqMan	Age, sex	–	0.336
Raptis	2007	Canada	European	Population	1359/1373	PCR-RFLP	Age, sex	147/1373	0.363
Campbell	2009	USA	Mixed	Population	1608/1968	PCR-RFLP	Age, sex, residence area, family history of CRC	193/1968	0.714
Whiffin	2011	UK	European	Population	10409/6965	PCR-RFLP	Age, sex, ethnicity, cancer free	358/6965	0.401

CRC, colorectal cancer; MSI, microsatellite instability; HWE, Hardy–Weinberg equilibrium; RFLP, restriction fragment length polymorphism.

**Figure 2:**
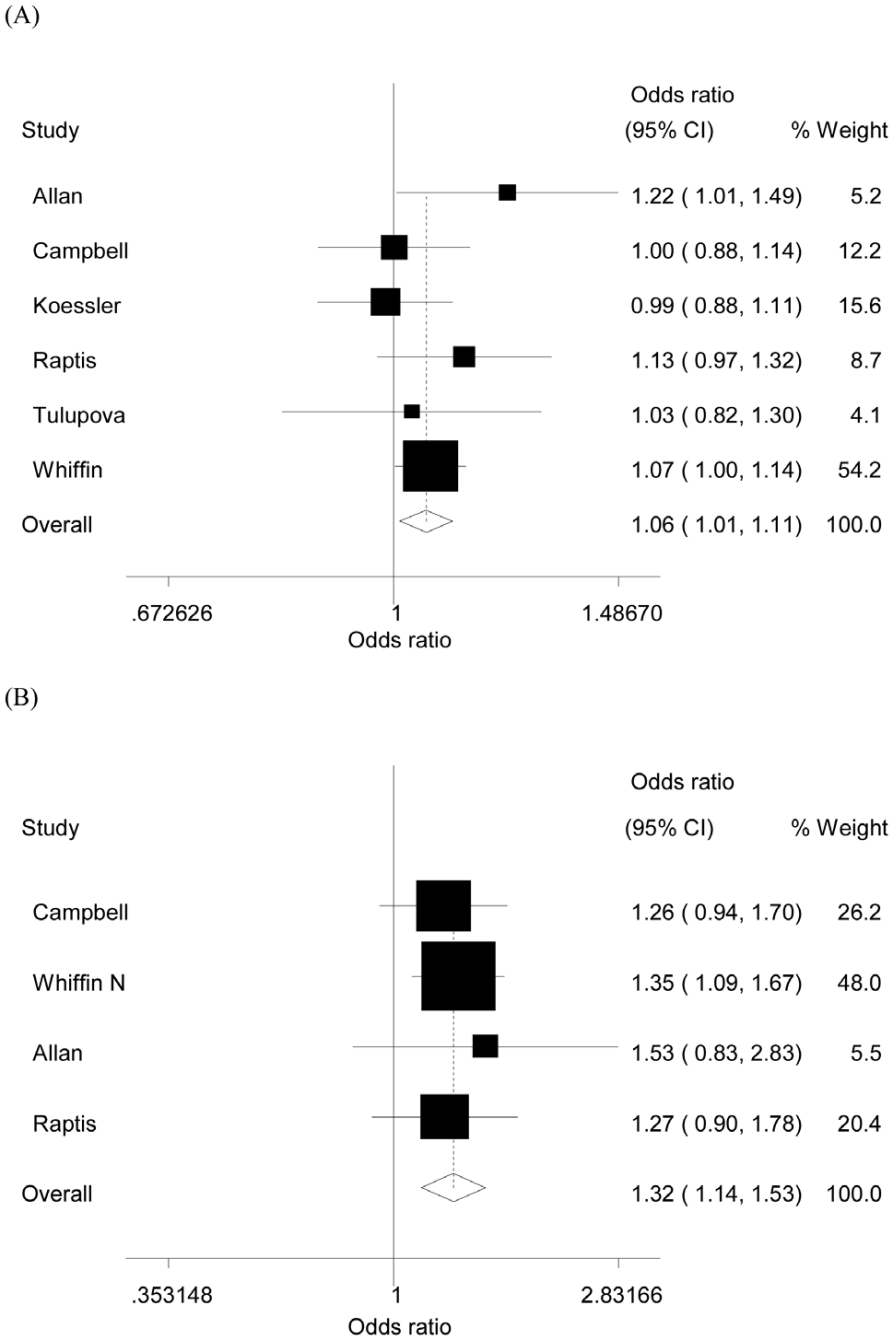
Forest plot of the risk of (A) CRC and (B) MSI-CRC associated with the *MLH1* -93G>A polymorphism (AA/AG versus GG). The areas of the squares reﬂect the study-specific weight (inverse of the variance). The diamonds represent the summary OR and 95% CI. The unbroken vertical line is at the null value (OR = 1.0).

**Table 2:** Meta-analysis of the effects of *MLH1* -93G>A polymorphism on risk of CRC.

Comparisons	OR	95% CI	*P*	*P* a
Total (n = 6)				
AA versus GG	1.08	0.97–1.20	0.171	0.881
AG versus GG	1.06	1.01–1.11	0.025	0.457
AA/AG versus GG	1.06	1.01–1.11	0.015	0.419
AA versus AG/GG	1.06	0.95–1.18	0.302	0.930
MSI status (n = 4)				
AA versus GG	2.52	1.94–3.28	<0.001	0.444
AG versus GG	1.29	1.10–1.52	0.002	0.216
AA/AG versus GG	1.45	1.24–1.68	<0.001	0.180
AA versus AG/GG	2.29	1.78–2.96	<0.001	0.620

a *P* value of Q test for heterogeneity test.

## Materials and Methods

### Identification and Eligibility of Relevant Studies

We searched the PubMed and EMBASE databases for all relevant articles. The last search update was June 1, 2012, using the search terms “*MLH1*,” “polymorphism,” and “colorectal cancer.” The search was limited to English-language articles. Additional studies were identified by a manual search of the references of the original studies. In the cases of multiple studies with the same or overlapping data published by the same investigators, we selected the most recent study with the largest number of subjects. Studies included in our meta-analysis met the following criteria: (a) evaluation of the *MLH1* -93G.>A polymorphism and CRC risk, (b) case-control design, and (c) sufficient published data for evaluation of the frequencies of various genotypes in cases and controls.

**Figure 3:**
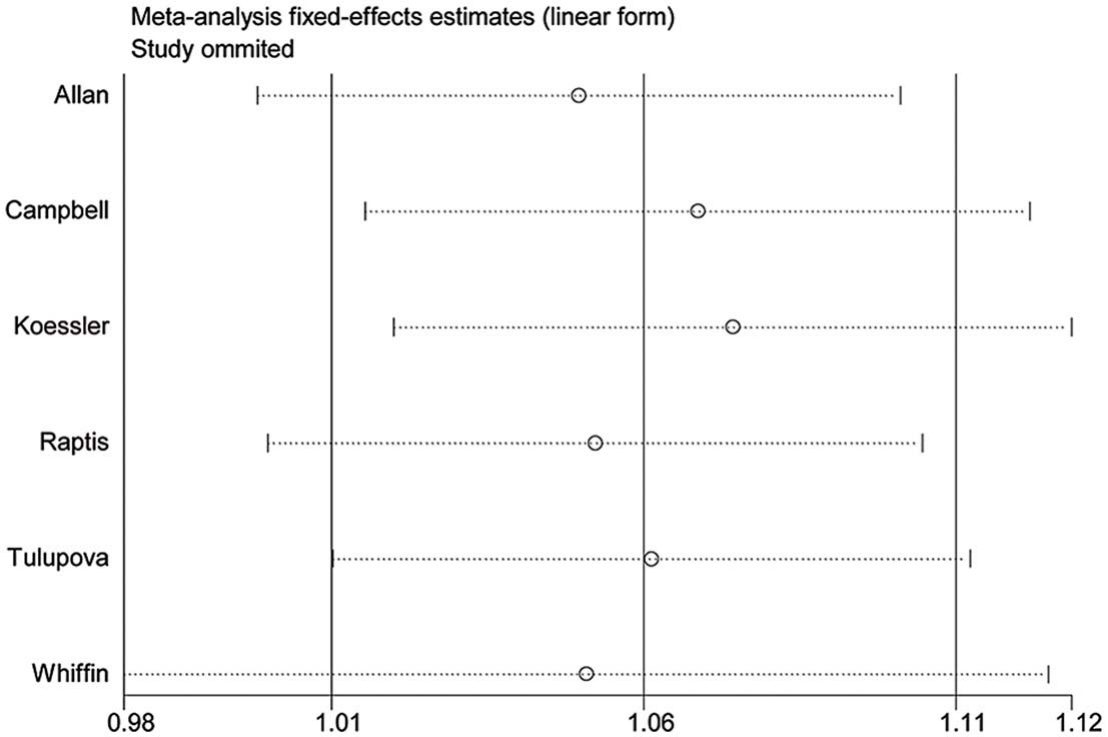
Analysis of the influence of AA/AG versus GG in the overall CRC meta-analysis. This figure shows the influence of individual studies on the summary OR. The middle vertical axis indicates the overall OR and the two vertical axes indicate the pooled OR when the left study is omitted from the meta-analysis. The two ends of the dotted lines represent the 95% CI.

**Figure 4:**
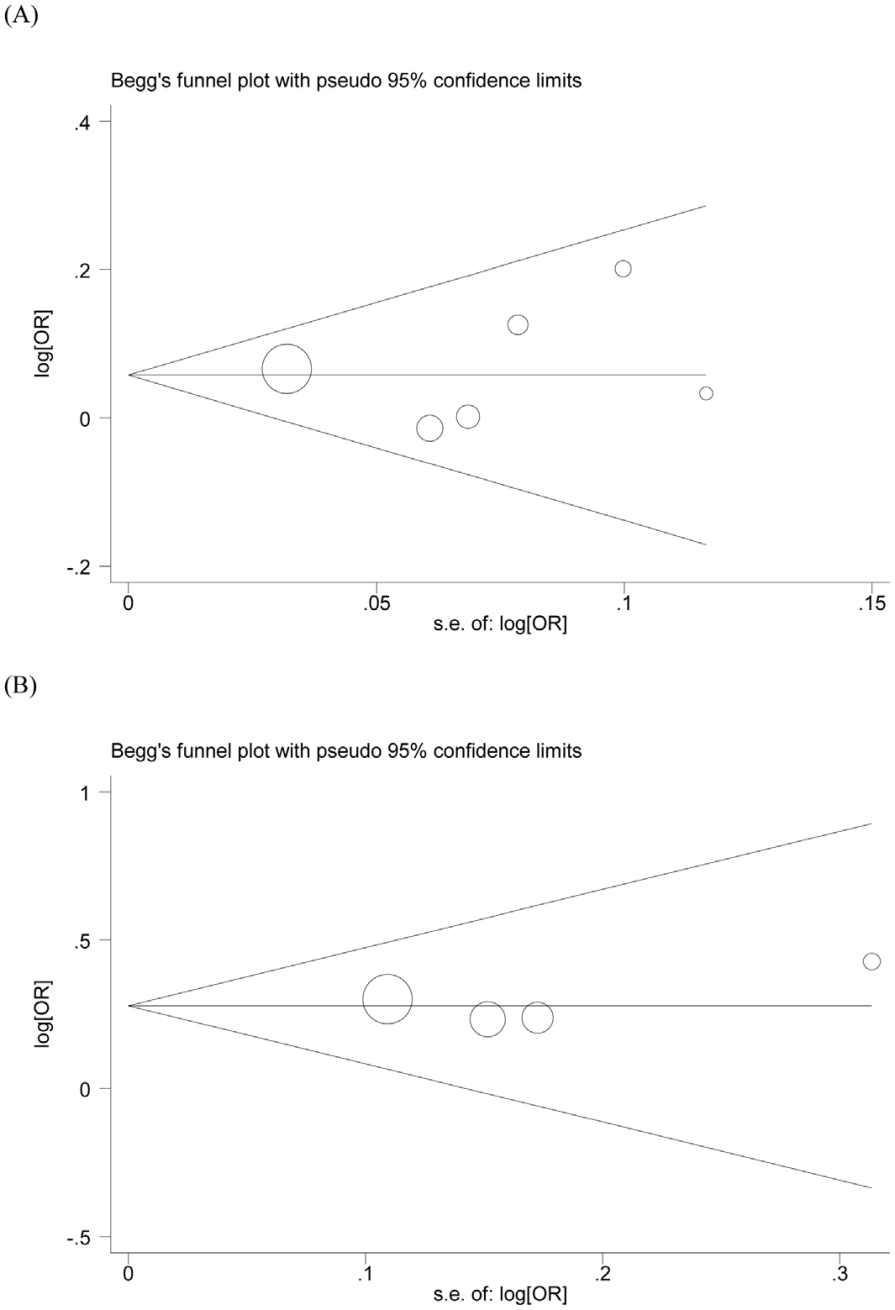
Publication bias test for the role of *MLH1* -93G>A polymorphism (AA/AG versus GG) in (A) CRC and (B) MSI-CRC. Each point represents a separate study of the indicated association. Log [or] is the natural logarithm of OR. Horizontal lines indicate the magnitude of the mean effect.

### Data Extraction

The following variables were extracted by two of the authors of the present paper (Ting Wang and Yang Liu). In the cases of conﬂicting evaluations, agreement was reached after a discussion. For each study, the following data were extracted: the first author’s surname, year of publication, country of origin, ethnicity of study subjects, source of controls, matching criteria, and sample size. Subjects were categorized as European, Asian, or mixed ethnicity. For studies that included subjects from different countries, data were extracted separately for each country group whenever possible.

### Statistical Analysis

Hardy-Weinberg equilibrium (HWE) was evaluated for each study using a goodness-of-fit chi-square test. Odds ratios (ORs) with 95% confidence intervals (CIs) were used to assess the strength of association between the *MLH1* -93G>A polymorphism and CRC risk. The pooled ORs were performed for co-dominant model (AA versus GG, or AG versus GG), dominant model (AA/AG versus GG), and recessive model (AA versus AG/GG). To assess the heterogeneity between the studies, a statistical test for heterogeneity was performed based on the Q statistic [12]. Where the studies were shown to be homogeneous with a *P*>0.10 for the Q test, the summary of OR estimate of each study was calculated using a fixed-effects model (the Mantel–Haenszel method) [13]. If there was significant heterogeneity, the random-effects model (the DerSimonian and Laird method) was used [14]. The stability of the results was assessed using sensitivity analyses, by randomly deleting combinations of cases and controls from different studies in the meta-analysis. Funnel plots and Egger’s linear regression test were used to assess publication bias [15]. The same measures were taken to estimate the degree of association between the *MLH1* -93G>A polymorphism and MSI-CRC risk. All analyses were performed with Stata software (version 8.2; StataCorp LP, College Station, TX), using two-sided *P* values.

## Results

### Study Characteristics

There were 312 published articles relevant to the search terms (Figure 1). By choosing additional filters, 285 of these papers were excluded (20 were not in English, 10 were not performed in humans, 246 were not performed on CRC, and 9 did not involve polymorphisms). By screening the titles and the abstracts, 21 of these studies were excluded. Only six articles were left for full publication review, and an additional two studies were included by manual searching of the reference lists of retrieved studies. Among these eligible full-text articles, one study was excluded for its small sample size in HNPCC [11]. Another was excluded because the controls had a history of CRC [16]. Finally, a total of six eligible studies involving 17,791 cases and 13,782 controls were included in the pooled analyses [6], [17]–[22]. The characteristics of selected studies are summarized in Table 1. The CRC cases were confirmed histologically or pathologically in most studies. Controls were usually matched with respect to age and sex. The distribution of genotypes in the controls was consistent with HWE in all studies.

### Quantitative Synthesis

As shown in Table 2, the *MLH1* -93G>A polymorphism was significantly associated with increased risk of CRC in two genetic models: AG versus GG (OR = 1.06, 95% CI = 1.01–1.11), and AA/AG versus GG (OR = 1.06, 95% CI = 1.01–1.11, Figure 2A) (each study weighting for each study was from 4.1% to 54.2%). In the stratified analysis of MSI status, similar increased risks were found (AA versus GG: OR = 2.52, 95% CI = 1.94–3.28;AG versus GG: OR = 1.29, 95% CI = 1.10–1.52; AA/AG versus GG: OR = 1.45, 95% CI = 1.24–1.68; AA versus AG/GG: OR = 2.29, 95% CI = 1.78–2.96, Figure 2B) (each study was weighed from 5.8% to 50.5%).

### Tests for Heterogeneity, Sensitivity Analyses, and Publication Bias

No significant heterogeneity was observed between studies during overall comparisons (Table 2). A single study was removed from meta-analysis each time to determine the inﬂuence of its individual data sets to the pooled ORs, and the corresponding pooled ORs were not materially altered (Figure 3). Begg’s funnel plot and Egger’s test were performed to assess publication bias. The shapes of the funnel plots did not reveal any evidence of obvious asymmetry in any of the models. Then, the Egger’s test was used to provide statistical evidence of funnel plot symmetry. The results did not show any evidence of publication bias for CRC (t = 0.29, *P* = 0.789, Figure 4A) and MSI-CRC (t = 0.63, *P* = 0.592, Figure 4B).

## Discussion

MMR genes are among the most important DNA repair genes [23]. Variations in MMR genes may alter predisposition to malignant tumors, especially CRC [24]. Previous studies have shown that the *MLH1* -93G>A polymorphism is associated with increased risk of CRC [6], [19], [22]. Because two transcription binding sites, NF-IL6 and GT-IIB, exist in this promoter region of the *MLH1* gene, the -93G>A polymorphism may reduce *MLH1* transcription and expression, thereby reducing overall DNA repair capability [25].

Several investigators have investigated the association between this polymorphism and risk of CRC, but the results have been inconclusive. In an analysis of 1,518 patients with CRC, Allan et al. demonstrated that the -93A variant was associated with a significantly increased risk of MLH1-deficient CRC detected by immunohistochemistry, though this polymorphism was not correlated with MSI-CRC [17]. Whiffin et al. found a significant difference in -93A allele distribution between a large number of CRC patients and control subjects [22]. However, Campbell et al. [6] and Koessler et al. [18] found that the -93G>A polymorphism was not associated with risk of CRC. To explain these conflicting results, a meta-analysis of six studies involving 17,791 cases and 13,782 controls was conducted to derive amore precise estimation of the association. Our results suggested that the -93G>A polymorphism was associated with increased risk of CRC among the studies populations. One biologically plausible mechanism underlying this association may be that the -93G>A substitution alters promoter function, and the -93A allele may be associated with reduced activity [26]. It is also possible that altered transcription factor binding in the *MLH1* promoter region causes promoter methylation and gene silencing [25], [27].

Although three similar meta-analyses have reported the existence of an association between the *MLH1* -93G>A polymorphism and the risk of CRC [22], [28], [29], they showed some different results. For instance, Whiffin et al. reported the pooled effect on the -93G>A polymorphism and CRC based on five case-control studies with a total of 14,121 CRC cases and 10,890 controls [22]. They provided more evidence an association between the -93G>A polymorphism and the risk of CRC. Pan et al. carried out a meta-analysis on all then-published case-control studies to estimate the overall cancer risk of the *MLH1* -93G>A polymorphism and showed that this polymorphism was not associated with increased risk of cancer. This null result was inconsistent with our results, possibly because the pooled data evaluated by these teams may have included incorrect information. For example, the controls included in the study by Chen et al. were all CRC patients without *MLH1* methylation, not individuals without a history of CRC. Therefore, it would be valuable if Pan et al. could provide a new more accurate estimation of the pooled results after excluding the data reported by Chen et al. [16]. Xu et al. performed a meta-analysis to assess the overall contributions of -93G>A and I219V polymorphisms in the *MLH1* gene to cancer susceptibility. They found that the -93G>A polymorphism was associated with increased risk of CRC, but the I219V polymorphism was not. The association between the -93G>A polymorphism and the risk of MSI-CRC could not be estimated. In this way, our meta-analysis provides an overview of the relevant studies and generates more exact pooled results of the associations between the -93G>A polymorphism and risk of CRC.

MSI is characterized by widespread changes in the length of short regions of repetitive DNA sequences, called microsatellites. A number of studies have reported associations between the -93G>A polymorphism and susceptibility to MSI-CRC. Our results showed that the -93G>A polymorphism could contribute to the risk of MSI-CRC. This was compatible with previous results. Raptis et al. showed the *MLH1* -93G>A variant allele to be associated with a higher risk of developing MSI-CRC than the wild-type allele [19]. Whiffin et al. also showed the effect on the -93G>A polymorphism and CRC risk to be confined to MSI-CRC, acting a marker for a somatic event [22]. There have been relatively few studies of CRC and microsatellite stability (MSS-CRC). In this way, the sample size for the -93G>A polymorphism and MSS-CRC was too small for meta-analysis. However, no significant association between the -93G>A polymorphism and MSS-CRC risk has been demonstrated in any previous study [6], [22].

This meta-analysis has limitations that must be acknowledged. First, the eligible studies included only published studies; so any preexisting publication bias will be reflected in our results, although the statistical data may not show it. Second, our lack of access to original data from the reviewed studies limited further evaluation of potential interactions. For example, the smoking status and die can help evaluate gene-gene and gene-environment interactions. Third, misclassifications of disease status and genotypes may also inﬂuence the results; many of the cases in several studies were not confirmed by pathology or other gold-standard methods. This is especially relevant to diversity within MSI status.

In conclusion, our meta-analysis suggests that the *MLH1* -93G>A polymorphism is associated with an increased risk of MSI-CRC. Large and well-designed epidemiological studies are warranted to validate our findings.
